# The chemical diversity and structure-based discovery of allosteric modulators for the PIF-pocket of protein kinase PDK1

**DOI:** 10.1080/14756366.2018.1553167

**Published:** 2019-01-03

**Authors:** Xinyuan Xu, Yingyi Chen, Qiang Fu, Duan Ni, Jian Zhang, Xiaolong Li, Shaoyong Lu

**Affiliations:** a Department of Pathophysiology, Key Laboratory of Cell Differentiation and Apoptosis of Chinese Ministry of Education, Shanghai Jiao Tong University, School of Medicine, Shanghai, China;; b Department of Orthopedics, Shanghai General Hospital, Shanghai Jiao Tong University, School of Medicine, Shanghai, China;; c Department of Orthopedics, Changhai Hospital, Naval Military Medical University, Shanghai, China

**Keywords:** PI3K, PDK1, allosteric modulators, allostery, orthosteric ligands, ATP binding site

## Abstract

Phosphoinositide-dependent protein kinase-1 (PDK1) is an important protein in mediating the PI3K-AKT pathway and is thus identified as a promising target. The catalytic activity of PDK1 is tightly regulated by allosteric modulators, which bind to the PDK1 Interacting Fragment (PIF) pocket of the kinase domain that is topographically distinct from the orthosteric, ATP binding site. Allosteric modulators by attaching to the less conserved PIF-pocket have remarkable advantages such as higher selectivity, less side effect, and lower toxicity. Targeting allosteric PIF-pocket of PDK1 has become the focus of recent attention. In this review, we summarise the current advances in the structure-based discovery of PDK1 allosteric modulators. We will first present the three-dimensional structure of PDK1 and illustrate the allosteric regulatory mechanism of PDK1 through the modulation of the PIF-pocket. Then, the recent advances of PDK1 allosteric modulators targeting the PIF-pocket will be recapitulated detailly according to the structural similarity of allosteric modulators.

## 1. Introduction

Phosphoinositide-dependent protein kinase-1 (PDK1) is a major regulator of the AGC family of kinases that phosphorylate and activate at least 23 related AGC protein kinases^1^, such as p70 S6 kinase (S6K)^2^^,^^3^, p90 ribosomal S6 kinase (RSK)^4^^,^^5^, serum and glucocorticoid-induced protein kinase (SGK)^6^^,^^7^, PKC isoforms^8^^,^^9^, protein kinase C-associated kinase 2 (PRK2)^10^, and PKB/AKT^11–13^. They can initiate tumorigenesis through the constitutive activation of kinases induced by oncogenic mutations^14–16^. Moreover, PDK1 plays a key role in the PI3K-AKT pathway, which is one of the most common deregulations in human cancers^17–20^. Thus, selective modulators of PDK1 may have utility as anti-cancer agents^21–24^.

PDK1 possesses three ligand binding sites: the substrate binding site, the catalytic ATP binding site, and the PDK1 Interacting Fragment (PIF) binding site^25^. The PIF-pocket, which is a hydrophobic surface pocket, has two functions: the recruitment of the downstream substrate kinases harbouring the hydrophobic motif (HM) and the stimulation of the intrinsic activity of PDK1^26^. Initially, a lot of efforts was devoted to the development of inhibitors targeting the ATP binding site which is relatively conserved in more than 500 protein kinases encoded by the human genome^27–35^. However, ATP-competitive inhibitors generally suffer from a low selectivity for PDK1, affecting other protein kinases unsatisfactorily and subsequently leading to potential side effects^36^. As an alternative, there is increasingly attractive in the discovery of non-ATP-competitive inhibitors of PDK1 in drug development^37^^,^^38^.

A possible strategy to develop non-ATP-competitive inhibitors is the design of selective allosteric modulators of PDK1 through binding to their allosteric sites, which are spatially and topographically distinct from its orthosteric, ATP binding site^39^^,^^40^. Allostery is a very efficient mechanism which regulates the function of biological macromolecules through the binding of an effector to an allosteric site distinct from the orthosteric, active site^41–47^. Compared to orthosteric ligands that bind to highly conserved orthosteric site, allosteric modulators, by targeting less conserved allosteric sites, offer several remarkable advantages such as greater selectivity, fewer side effects, and lower toxicity^46^^,^^48–50^. Therefore, allostery has been a crucial strategy for the development of non-ATP-competitive kinase inhibitors^51^^,^^52^.

The PIF-pocket, which is distant from the ATP binding site, can act as an allosteric site of PDK1^53^^,^^54^. As a result, molecules directed to the PIF-pocket have much higher selectivity for PDK1 against its homologous kinases^55^. In contrast to complete inhibition of enzyme activity by ATP-competitive inhibitors, targeting allosteric sites may endow modulators with different functions such as inhibition or activation of protein kinases. For example, several allosteric modulators bound to the PIF-pocket can act as inhibitors or activators in the regulation of PDK1 function^56^. In addition, allosteric modulators do not compete with the ATP-competitive inhibitors. Indeed, they can work together in an individual protein^57^. Although the development of allosteric modulators still faces a few challenges such as low binding affinities and poor solubility, it represents a novel strategy for drug discovery due to their remarkable advantages^58–61^. In recent years, the discovery of PDK1 allosteric modulators has experienced an upsurge with a significant increase in their number.

In this review, we summarise the current advances in the development of PDK1 allosteric modulators. We will first present the crystal structure of PDK1 and the binding mechanisms of PDK1 allosteric modulators briefly. Then, the recent discovery of PDK1 allosteric modulators targeting the PIF-pocket will be elucidated detailly, which may help the design of PDK1 allosteric modulators with an improved potency and selectivity for the treatment of human diseases.

## 2. The three-dimensional structure of PDK1

**Figure 1.**
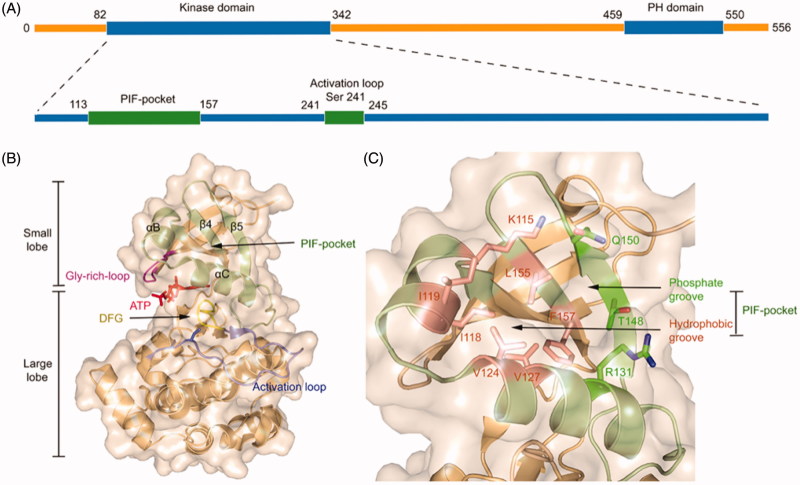
(A) The general structure of the peptide chain of PDK1. (B) Structural features of the catalytic core of PDK1. The PIF-pocket is shown in pale green, the activation loop in slate, the Gly-rich-loop in magenta, the DFG in yellow, and ATP in red. (C) Structural features of the PIF-pocket of PDK1. The residues that form the phosphate groove are shown in green, while the other residues that form the hydrophobic groove are shown in salmon.

PDK1 is one of the most ancient and conserved protein kinases, which belongs to the AGC protein kinases. Unlike other AGC kinases, the catalytic domain of PDK1 does not possess a HM that is related to autophosphorylation^62^. The full-length of PDK1 protein contains 556 amino acid residues that comprise an N-terminal serine-threonine kinase domain and a C-terminal pleckstrin homology (PH) domain (Figure 1(A))^63^^,^^64^. Within the kinase domain, the polypeptide chain can be further subdivided into two lobes where the C-terminal lobe (C-lobe) is much larger than the N-terminal lobe (N-lobe) (Figure 1(B))^65^. The activation loop (T-loop) is located between the large C-lobe and the helix αC of the N-lobe and positioned after a conserved DFG motif. Notably, phosphorylation of residue Ser241 on the T-loop is strictly required for PDK1 kinase activity^66^^,^^67^. Between the two lobes is the ATP binding site whose roof is formed by the Gly-rich-loop^68^. In the small N-lobe, the αB and αC helixes and the adjacent β4 and β5 strands form a ∼5 Å deep pocket, named the PIF-pocket, which enables to recognise the phosphorylated HM of substrate kinases^69^. The PIF-pocket consists of a separate hydrophobic groove and a positively charged phosphate-binding groove (Figure 1(C)). Previous studies have shown that the hydrophobic groove rather than the phosphate groove is the determinant for the phosphorylation of substrate kinases by PDK1, and the phosphate groove only serves a key factor in maximising phosphorylation of PDK1 substrates^70^. In the PIF-pocket, residue Leu155 on the β-sheet 5 is located at the centre and other residues such as Lys115, Ile118, and Ile119 on the αB helixes and Val124 and Val127 on the αC helixes wrap the PIF-pocket. When individual residues Lys115, Ile119, and Glu150 were mutated into Ala, the affinity of each PDK1 mutant to its substrates was reduced. Previous studies have confirmed that residue Leu155 is essential for the ability of PDK1 to interact with the HM of substrates through the PIF-pocket. Interestingly, when Leu155 was mutated by Ala, it increased the activity of PDK1, which may be instructive for the design of allosteric activators of PDK1^71^.

## 3. Binding mechanisms of PDK1 allosteric modulators

It is well-established that the activity of protein kinases is usually regulated by the phosphorylation of T-loop residues, which triggers conformational changes in their catalytic domain and subsequently facilitates the entry of substrates and ATP to the kinase domain^72–74^. The optimal activation of AGC kinases family requires both phosphorylation of residues located at the T-loop and HM^75^^,^^76^. In the active state, PDK1 is identified as the primary kinase to phosphorylate T-loop of other AGC kinases^77^^,^^78^. Except PKB, almost all of the PDK1 substrates must dock their own C-terminal HMs into the PIF-pocket of PDK1 for phosphorylation and activation^79–81^. In the meanwhile, the resulting docking can enhance the catalytic activity of PDK1 through an allosteric communication from the allosteric PIF-pocket to the orthosteric ATP binding site^82–84^.

**Figure 2.**
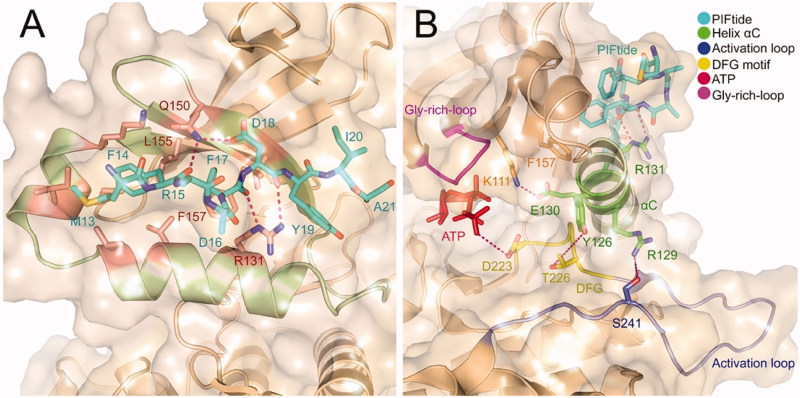
(A) Ribbon representation of the X-ray structure of PDK1-PIFtide complex (PDB ID: 4RRV). The residues that form the PIF-pocket are shown in salmon, while the other residues from PIFtide are shown in cyan. (B) Ribbon representation of the connection of the helix αC with the activation loop, DFG, and Gly-rich-loop. The helix αC and related residues are shown in green, DFG and related residues are shown in yellow, activation loop and related residues are shown in slate, and Gly-rich-loop is shown in magenta.

The above-phosphorylated residues that bind to the PIF-pocket can sometimes be replaced by the acidic residues of short peptides such as PIFtide^85^. PIFtide is a 24-amino acid polypeptide derived from the PDK1 substrate PRK2, which can stimulate PDK1 activity toward a short peptide substrate but disrupt recruitment and phosphorylation of the full-length substrates S6K and SGK^86–88^. PIFtide is the most effective HM peptide toward PDK1 with a K_d_ of 4 3 ∼ 90 nM and a 7-fold enhanced activation for PDK1. Sadowsky *et al.* previously elucidated the orientation of PIFtide in the PIF-pocket of PDK1 and explored the subtle relationship between PIFtide localisation and kinase activation through disulphide trapping approach^56^. Then, Rettenmaier *et al*. determined the first high-resolution crystal structure of the PDK1-PIFtide complex^57^ (Figure 2(A)). This complex illustrates the interaction of PIFtide with the PIF-pocket and the mechanism by which conformational changes at the PIF-pocket are transmitted to the ATP binding site. There are two obvious interactions in the complex. One is the hydrogen bonding interaction that is formed by the conserved negatively charged residue Asp18 of PIFtide with the residue Gln150 of PDK1 and the other is the salt bridge interaction that is formed by the residue Asp16 of PIFtide with the Arg131 of PDK1. In addition, the side chain aromatic rings of the conserved residues Phe14, Phe17, and Tyr19 of PIFtide occupy three hydrophobic sub-pockets, respectively. Residues Met13, Ile20, and Ala21 of PIFtide protrude the PIF-pocket. The docking of PIFtide to the PIF-pocket not only causes the above conformational changes in the pocket, but these changes are in turn transmitted to the ATP binding site through the critical component of the helix αC (Figure 2(B)). The interaction of PIFtide with the residue Arg131 causes a slight movement of the helix αC, which further leads to partial changes of residues Arg129, Tyr126, Glu130. The hydrogen bonding interactions between these residues with the residue Ser241 on the T-loop, Thr226 on the DFG motif, and Lys111 adjacent to the Gly-rich-loop conduct the allosteric signal to the ATP binding site. To reveal the relative energetic contribution of each residue from PIFide, alanine scanning mutagenesis method was applied to identify the binding energy hotspots. Five hotspot residues of PIFtide were then identified, including Met13, Phe14, Phe17, Asp18, and Tyr19.

Since phosphorylation can be mimicked by the docking of PIFtide, it is reasonable that non-peptide small molecules may also mimic the interactions required to trigger corresponding conformational changes. Engel *et al*. confirmed that small molecules can provide all the necessary requirements for conformational changes, resulting in the enhanced activation of PDK1^85^. Stroba *et al*. obtained the first co-crystal of the allosteric small molecule bound to the PIF-pocket of PDK1^89^. By virtue of fluorescence-based assay and deuterium exchange experiments, Hindie *et al*. observed the local changes at the PIF-pocket and the conformational changes at the T-loop of the ATP binding site, which are induced by the binding of allosteric modulators at the PIF-pocket^90^. Further experiments indicated that both the hydrophobic interactions formed between the aromatic rings of allosteric modulators and the PIF-pocket and the ionic interactions formed between the carboxyl group of allosteric modulators and residues Thr148 or Arg131 from the PIF-pocket played critical roles in the binding of allosteric modulators.

PIF-pocket can recruit the downstream substrate kinases harbouring the HM and stimulate the intrinsic activity of PDK1. Therefore, when an allosteric modulator binds to the PIF-pocket, it changes the intrinsic activity of PDK1 by competition with the HM peptide of natural substrate kinases. The previous studies showed that blocking of PIF-pocket was the primary effect by these binding molecules, although such molecules may be allosteric activators of PDK1 *in vitro* essay^55^^,^^91^. Furthermore, the activation of most downstream kinases of PDK1 requires docking of substrate kinases to the PIF-pocket expect for AKT^92^. As a result, regardless of whether an allosteric modulator activates or inhibits PDK1 *in vitro*, a large probability behaves as an inhibitor for the entire signalling pathway in vivo. Here, we only focus on the allosteric modulators targeted to the PIF-pocket with their effects on PDK1 itself *in vitro*, regardless of their effects on the signalling pathway.

## 4. Allosteric activators of PDK1

**Figure 3.**
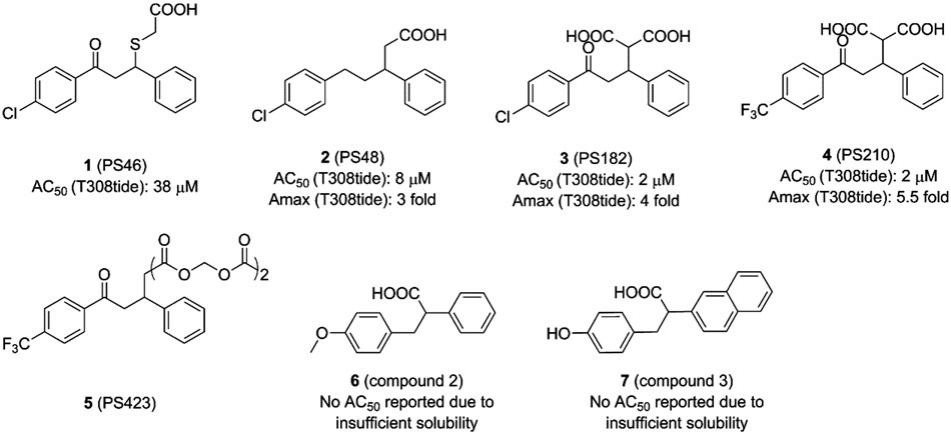
Structure and biochemical characterisation of diary carboxylic acid derivatives. AC_50_: compound concentration required for 50% of maximum activation of PDK1; Amax: maximum activation of PDK1 compared to DMSO control (=100%); T308tide: a synthetic substrate peptide in the radioactivity-based kinase assay that does not interact with the PIF-pocket but could be phosphorylated by PDK1.

**Figure 4.**
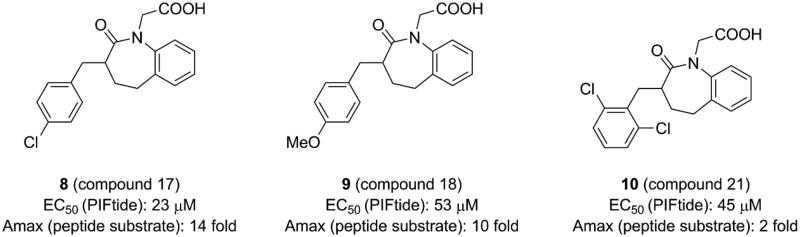
Structure and biochemical characterisation of benzoazepin-2-one derivatives. EC50: compound concentration is required to result in 50% decrease of HTRF signal in a HTRF competition assay, representing the ability of compounds to displace PIFtide peptide; Amax: maximum activation of PDK1 compared to DMSO control (=100%).

**Figure 5.**
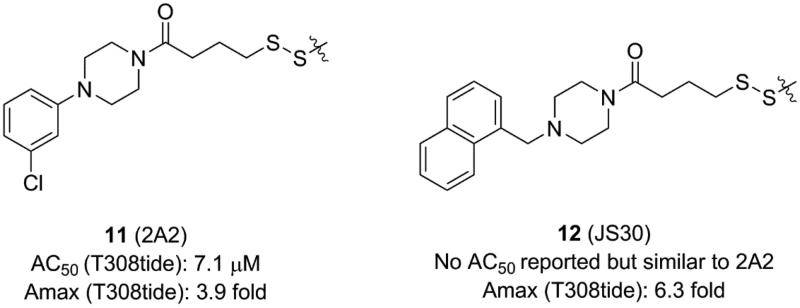
Structure and biochemical characterisation of disulphide derivatives.

**Figure 6.**
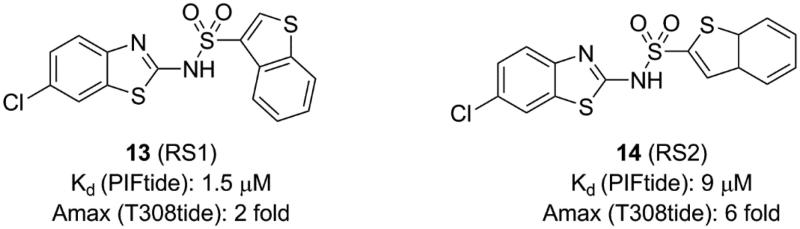
Structure and biochemical characterisation of diaryl sulphonamide derivatives. Kd: the ability of compounds to displace PIFtide in a FP competitive binding assay.

**Figure 7.**
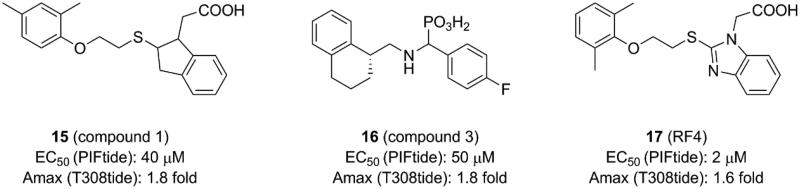
Structure and biochemical characterisation of benzimidazole derivatives. EC50: compound concentration is required to result in 50% displacement of the fluorophore-labeled PIFtide from the PIF-pocket in a FP competitive binding assay.

**Figure 8.**
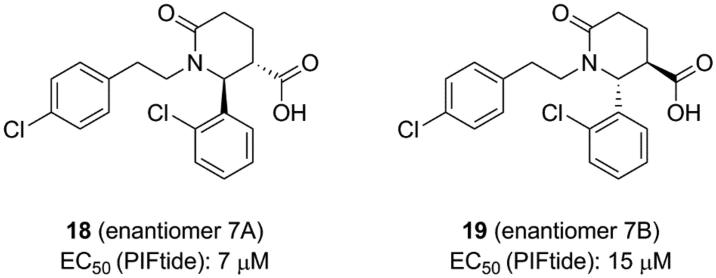
Structure and biochemical characterisation of oxypyridine derivatives. EC_50_: concentration that resulted in 50% displacement of the PIFtide from the PIF-pocket in an AlphaScreen interaction-displacement assay.

In recent years, a major stride has been made in the discovery of PDK1 allosteric activators. Based on the identified allosteric activators, we classify these molecules into six classes according to their similarity of chemical structures, including diary carboxylic acid derivatives (Figure 3), benzoazepin-2-one derivatives (Figure 4), disulphide derivatives (Figure 5), diaryl sulphonamide derivatives (Figure 6), benzimidazole derivatives (Figure 7), and oxypyridine derivatives (Figure 8). We focus on the detailed PDK1-modulator interactions at the PIF-pocket for the best potent molecule from each class.

### 4.1. Diary carboxylic acid derivatives

Based on the pharmacophore model defined by the C-terminal HM of PKA that possesses key residues Phe347 and Phe350, Engel *et al*. in silico screened a chemical library consisting of 60,000 low-molecular-weight molecules and then tested potential molecules in vitro^85^^,^^93^. Finally, PS46 (**1**, Figure 3) was identified for its high activation level with a low AC_50_ (AC_50_=concentrations that gives 50% of maximum activation). Site-directed mutagenesis at the centre of PIF-pocket eradicated the binding of **1** to PDK1, strongly supporting the notion that PIF-pocket is a bona fide target of this molecule. Similarly, **1** disrupted the interaction between PDK1 and PIFtide in a surface plasmon resonance (SPR) assay. Consistent with above phenomena, the phosphorylation of substrates S6K and SGK by PDK1, which are required to dock into the PIF-pocket of PDK1, was blocked by **1**. Most importantly, when residue Arg131 was mutated by Met or Ala to lose its positive charge, the catalytic activity of PDK1 mutant could not be increased by **1**. But when Arg131 was mutated by Lys to retain its positive charge, the catalytic activity of PDK1 mutant was not affected. Moreover, when the carboxyl group of **1** was converted to an ester group, the catalytic activity of PDK1 showed no change. Further computational docking revealed that the salt bridge interaction between the carboxylic acid group of **1** and the residue Arg131 of PIF-pocket is essential for the allosteric activation of PDK1. Finally, the selectivity of this molecule was proven to be excellent by testing on other AGC kinases. Taken together, **1** is the first reported small molecule which can enhance the catalytic activity of PDK1 by targeting the allosteric PIF-pocket.

**Figure 9.**
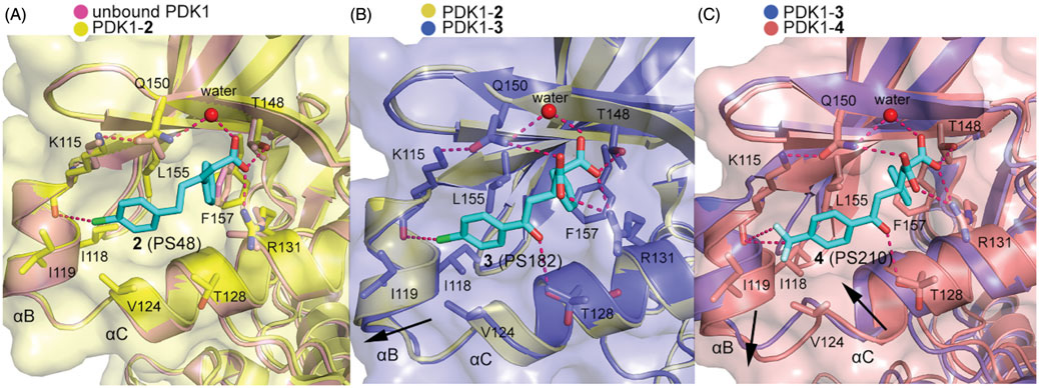
(A) Comparison of the X-ray structure of unbound PDK1 (pink; PDB ID: 3HRC) with the X-ray structure of PDK1-**2** complex (pale yellow; PDB ID: 3HRF). (B) Comparison of the X-ray structure of PDK1-**2** complex with the X-ray structure of PDK1-**3** complex (slate; PDB ID: 4AW0). (C) Comparison of the X-ray structure of PDK1-**3** with the X-ray structure of PDK1-**4** complex (salmon; PDB ID: 4AW1).

**1** contains a chiral centre which requires to evaluate the contribution of each enantiomer to the total activity. The sulphide group also has potential oxidisation and is prone to arise retro-Michael reactions. By replacing the chiral centre with a double bond and retaining the three hybridised C-atoms in the chain to connect the two benzene rings, Stroba *et al*. synthesised PS48 (**2**, Figure 3) that showed a 4-fold lower AC_50_ than **1**^89^. Importantly, the crystal structure of **2** bound to PDK1 was determined. The comparison between the cocrystal structure of PDK1-**2** complex with the structure of PDK1 alone reveals details of the allosteric mechanism by **2** (Figure 9(A)). The PDK1-**2** complex shows that the carboxylic acid group of **2** interacts with residues Arg131 and Thr148 of PDK1 through salt bridge and hydrogen bonding interactions and with residue Gln150 via water-mediated hydrogen bonding interaction. Residue Leu155 separates the hydrophobic groove into two sub-pockets that are occupied by the two benzene rings of **2**. Especially, the right benzene ring forms edge-to-face CH-π interactions with residue Phe157 and the chlorine atom of the benzene ring on the left forms a halogen bond with residue Lys115. In addition, the residues Arg131, Glin150, and Phe157 rotate about 90° and the rotation of residue Lys115 reaches 180°. The resulting movement of these residues enlarges the depth of the PIF-pocket and stabilises the helix αC. The stabilisation of the helix αC limits the conformational shift of the T-loop, Gly-rich-loop, and DFG motif, thereby increasing the rigidity of the ATP binding site. Furthermore, Hindie *et al*. verified the molecular mechanism of allosteric changes induced by **2** through fluorescence-based assay and deuterium exchange experiments^90^. Overall, **2** binding caused local changes in the PIF-pocket, which allosterically triggered conformational changes at the T-loop and the ATP binding site. Through analysis of structure-activity relationships (SAR) of **2**, the minimal structural requirements for an allosteric modulator toward PDK1 were proposed: two aromatic groups linked by an aliphatic chain, a side chain with a free carboxyl group, and a V-shaped overall conformation of the aryl rings.

To further improve potency of **2**, Wilhelm *et al*. optimised this activator by retaining the ketone group of **1** and introducing the shorter carboxyl side chain from **2**^55^, because both the keto group and the carboxyl group play a key role in the binding affinity of allosteric activators by offering additional ionic interactions. Thus, the two carboxyl groups were introduced in the optimisation, yielding PS182 (**3**, Figure 3) with an AC_50_ value of 2.5 μM and a 4-fold enhanced activation for PDK1. Although there was an improvement of affinity through a dicarboxyl moiety, optimisation of the ring substituents may achieve a better result. Obviously, this conjecture is true for the identification of PS210 (**4**, Figure 3) where the chlorine moiety in the benzene ring of **3** was replaced by a trifluoromethyl group. It showed that **4**, with an AC_50_ value of 2 μM, displayed a 5.5-fold enhanced activation for PDK1. Notably, both **3** and **4** increased the thermal stability of PDK1 whereas **2** had almost no effect measured by differential scanning fluorimetry (DSF) assays.

**Figure 10.**
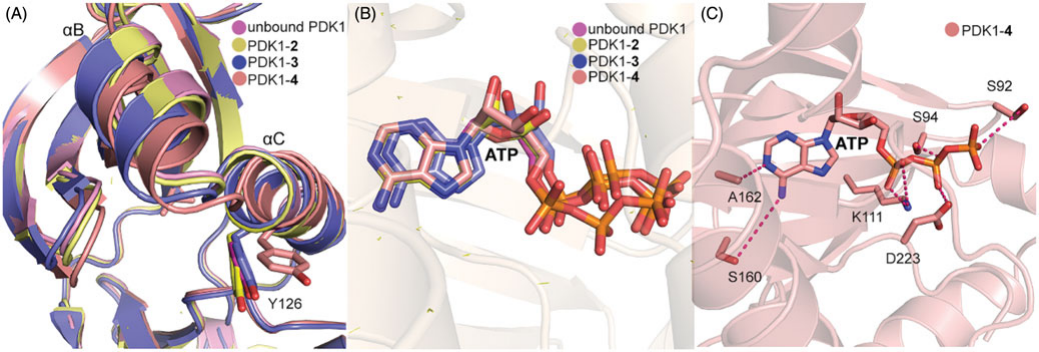
(A) Comparison of the helix αB and helix αC of unbound PDK1 (magenta), PDK1-**2** complex (pale yellow), PDK1-**3** complex (slate), PDK1-**4** complex (salmon). (B) Comparison of the ATP of unbound PDK1 (magenta), PDK1-**2** complex (pale yellow), PDK1-**3** complex (slate), PDK1-**4** complex (salmon). (C) Cartoon/surface representations of the interaction between ATP with the ATP binding site of PDK1-4 complex.

Both **3** and **4** were co-crystallized by soaking each molecule with PDK1 crystal. As shown in Figure 9(B), the overall binding mode of PDK1-**3** complex was very similar to that of PDK1-**2** complex. Two hydrophobic sub-pockets are also occupied by the benzene rings of **3**, and the CH-π interactions between the right benzene ring with residue Phe157 are remained. However, the second carboxyl group of **3** forms an additional salt bridge with residue Arg131 of the PIF-pocket, and directly interacts with residue Gln150. It is also noteworthy for the interaction between the residue Thr128 of PDK1 and the keto group of **3**. Deep in the pocket, **3** causes a downward movement of the helix αB, which can drive the change of the Gly-rich-loop of the ATP binding site. This conformational change leads to reshape the phosphate groups of ATP. As shown in Figure 9(C), aside from the above interactions, the trifluoromethyl substituent of **4** forms additional hydrophobic interactions with the residues of helix αB such as Val124, Ile118 and Ile119. These interactions cause the helix αB to move further by 1.1 Å in a direction perpendicular to the first motion. Comparing the helix αB and the helix αC in the crystal structures of PDK1-**2**, PDK1-**3**, PDK1-**4**, and unbound PDK1, it can be found that the helix αB gradually moves down with the increase of PDK1 activation factor, but the displacement of the helix αC seems to be irregular (Figure 10(A)). The allosteric signal is transmitted to the ATP binding site through the Gly-rich loop. Further comparison of ATP structure in the four structures shows that the β, γ-phosphates of ATP in the PDK1-**4** complex rotated largely (Figure 10(B)). The resulting movement increases the interaction of ATP with the residues Asp223, Lys111 and Ser94 (Figure 10(C)). Overall, the affinity of allosteric activators is proportional to the number of contacts that are formed by activators with the PIF-pocket. Additionally, the substituents on the left benzene ring have a further optimised space to exert more influence on the helix αB.

The diary carboxylic acid derivatives have poor permeability due to the negative charge of the carboxyl group. To improve the permeability and explore the pharmacologic effects of these modulators more efficiently, carboxyl groups were replaced by ester groups, yielding PS423 (**5**, Figure 3). This molecule could be cleaved to produce the homologous diary carboxylic acid derivatives by cellular esterases and was identified as a prodrug of **4** that can readily permeate cell membrane. Further experiments revealed that **5** did not affect the activity of PDK1 *in vitro*, which once again confirmed the necessity of carboxylic acid groups for the activation of PDK1.

The fragment screening method is an orthogonal complement of high throughput screening for searching ligands for biomacromolecules. Since fragments are limited to low molecular weights, the efficient sampling of chemical space with ∼10 K fragments or even less can be accomplished. Moreover, the screened fragments are usually hydrophilic and can be easily chemically modified, which is more suitable as a starting point for drug development. Stockman *et al.* developed an NMR fragment screening approach for identifying small molecules binding to both the ATP and the PIF-pocket of PDK1^94^. Then, by means of saturation transfer difference (STD) NMR experiments, the selected molecules can be distinguished between binding at the ATP binding site and at the allosteric PIF-pocket. Through screening, a fragment library of 10,000 diverse molecules, compounds 2 (**6**, Figure 3) and 3 (**7**, Figure 3) bound to the allosteric PIF-pocket were obtained. The two molecules were inactive at 313 μM in the Kinase-Glo assay, but they did not show any inhibition of PDK1. However, they showed 33 ± 13% and 30 ± 12% activation using the Calliper assay (a kinase activity assay through measuring the incorporation of a phosphate into a fluorescent tagged substrate peptide), respectively, compared to the control reaction at the 313 μM. However, it is difficult to determine AC_50_ values of both molecules owing to their poor solubility. Interestingly, the chemical structures of two molecules resemble diary carboxylic acid derivatives. All of them share one negatively charged carboxyl group and two hydrophobic benzene rings. The difference is that the length of the linker connecting the two aromatic rings is different. The flexibility of the longer carbon chain may explain the increased activity of **1** compared to both **6** and **7**. However, more specific allosteric details require co-crystallization of both **6** and **7** with PDK1.

### 4.2. Benzoazepin-2-one derivatives

Wei *et al.* analysed the above allosteric activators of PDK1 acquired by virtual screening and NMR-based fragment screening^95^. All structures of these molecules contain a negatively charged carboxylate and two aromatic hydrophobic groups. The carboxylate mimics the phosphate group of the substrate kinases and the effect of the two benzene rings are very similar to the phenylalanine residues of the HM. Based on this evidence, a suite of benzoazepin-2-one derivatives targeting the PIF-pocket of PDK1 were designed *de novo* through virtual docking. After in silico evaluation and further SAR exploration by using **1** as the positive control with an EC_50_ value of ∼133 μM, three excellent molecules, compound **17** (**8**, Figure 4) with an EC_50_ value of ∼23 μM, compound **18** (**9**, Figure 4) with an EC_50_ value of ∼53 μM, and compound **21** (**10**, Figure 4) with an EC_50_ value of ∼45 μM, were obtained.

Benzoazepin-2-one derivatives were designed using diary carboxylic acid derivatives as the template, so there are many similarities of chemical structures between them such as two benzene rings and one carboxylic acid. The difference is that the original hydrophobic chain in the diphenylpropionic acid derivatives is replaced by the benzoazepin-2-one scaffold. This substitution is beneficial for binding. First, the rigid benzodiazepine replaces the flexible hydrophobic chain to aid in the orientation of the benzene ring and the carboxylic acid. Then, the extra ketone group was designed, supposing that it may form an additional interaction with the residue Thr128 of PIF-pocket such as **4**. These could explain the higher affinity of **8**, **9** and **10** compared to **1**. Unexpectedly, **9** and **10** have the similar affinity, but show different levels of PDK1 activation. However, the co-crystal structures of these small molecules with PDK1 are still unavailable and the detailed mechanisms are unclear. Further research of the activation mechanism by benzoazepin-2-one derivatives is required to ascertain.

### 4.3. Disulphide derivatives

Sadowsky *et al.* developed a disulphide trapping method that allows small molecules to interrogate allosteric sites of protein kinases and subsequently assesses the effects of binding on protein structure and function^56^^,^^96^. Initially, they prepared six PDK1 Cys mutants in which residues at six positions around the PIF-pocket were mutated by a cysteine (K115C, I119C, V124C, R131C, T148C, and Q150C). A small library consisting of 480 disulphide fragments was screened and each mutant trapped a series of distinct disulphide fragments which showed a good selectivity.

**Figure 11.**
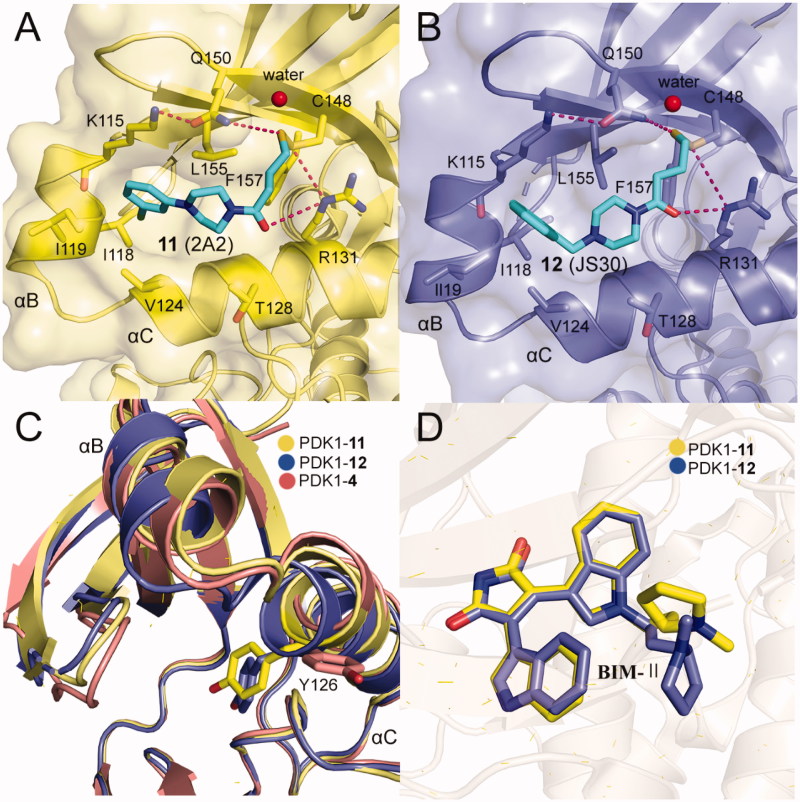
(A) Cartoon/surface representations of the interaction between **12** with the PIF-pocket of PDK1 (pale yellow; PDB ID: 3ORZ). (B) Cartoon/surface representations of the interaction between 12 with the PIF-pocket of PDK1 (slate; PDB ID: 3OTU). (C) Comparison of the helix αB and helix αC of PDK1-**11** complex, PDK1-**12** complex and PDK1-**4** complex (salmon). (D) Comparison of the BIM-IIof PDK1-**11** complex and PDK1-**12** complex.

The best fragment is 2A2 (**11**, Figure 5) with an EC_50_ value of ∼7.1 μM, which is special to the mutant PDK1^T148C^ and enhanced the activation of PDK1^T148C^ by about 3.9-fold. Optimisation of **11** resulted in JS30 (**12**, Figure 5), which achieved a 6.3-fold enhanced activation of PDK1^T148C^. The activation of PDK1 by **12** was slightly higher than the previously best molecule **4** which activated PDK1 by 5.5-fold. However, the activated ability of the three molecules toward PDK1 is different, so comparison of their cocrystal complexes is useful in the exploration of the mechanism of allosteric activation (Figure 11(A) and Figure 11(B)). The binding modes of the two disulphide molecules **11** and **12** at the PIF-pocket are similar to that of **4**. The remarkable difference is that the sulphur atom of **11** and **12** replaces the carboxylic acid group of **4** to form interactions with residues Arg131, Cys148, and Gln150. Especially, the disulphide bond formed by **11** and **12** with the mutated residue Cys148 is stronger than the corresponding hydrogen bond in the PDK1-**4** complex. This may be why **11** and **12** have a reasonable affinity for the PIF-pocket. In addition, since there were no benzene rings at the right end of **11** and **12**, the conformation of the critical residue Phe157 at the PIF-pocket does not undergo changes. Comparison of the conformational changes of the PIF-pocket shows that the helix αB has moved downward and the pyrrole ring of ATP-competitive ligand bis-mercaptomaleimide-II (BIM-II) has rotated about 90°in the **12**-bound PDK1 compared to the **11**-bound PDK1 (Figure 11(C) and Figure 11(D)). This is consistent with the diary carboxylic acid derivatives by which the downward movement of the helix αB is positively correlated with the protein activity.

### 4.4. Diaryl sulphonamide derivatives

Different from the covalent allosteric activators acquired through the disulphide trapping method, Rettenmaier *et al.* identified the noncovalent allosteric activators targeting the PIF-pocket of PDK1 by the site-directed chemical screen^57^^,^^97^. Fluorescence polarisation (FP) competitive binding assay was used to identify molecules that can disrupt the interaction between PIF-pocket and PIFtide. Initially, 154,000 molecules were screened at a single dose through the FP assay. Then, 1280 molecules in dose-response mode were further selected for testing using the FP and SPR assays. The top 10 hits were repurchased for kinase activity assay, and a diaryl sulphonamide derivative with the most potent activity was identified. To further optimise this parent molecule, a series of iterative synthesis was conducted. Testing by FP, SPR, and kinase activity assays, the regioisomers RS1 (**13**, Figure 6) and RS2 (**14**, Figure 6) with a K_d_ of ∼1.5 and ∼9 μM were obtained, respectively. Moreover, **13** and **14** stimulated PDK1 activity toward a short peptide substrate by 2-fold and 6-fold, respectively.

**Figure 12.**
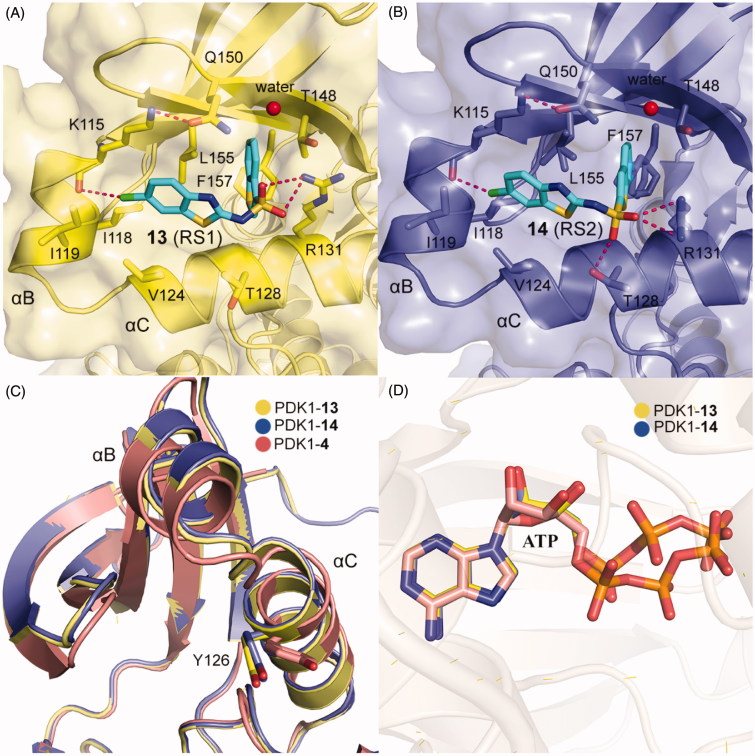
(A) Cartoon/surface representations of the interaction between **13** with the PIF-pocket of PDK1 (pale yellow; PDB ID: 4RQK). (B) Cartoon/surface representations of the interaction between **14** with the PIF-pocket of PDK1 (slate; PDB ID: 4RQV). (C) Comparison of the helix αB and helix αC of PDK1-**13** complex, PDK1-**14** complex and PDK1-**4** complex (salmon). (D) Comparison of the ATP of PDK1-**13** complex, PDK1-**14** complex, and PDK1-**4** complex.

Compared with diary carboxylic acid derivatives, **13** and **14** also had excellently biochemical characterisation, but the chemical structures of **13** and **14** were different from that of diary carboxylic acid derivatives. First of all, the benzene rings at both ends are replaced by the benzo heterocycles. With reference to previous benzoazepin-2-one derivatives, this is expected to increase the rigidity of the entire molecule and thereby aids in the orientation of the functional groups. Then, the substitution of a sulphonamide group with a carboxylic acid group greatly improves the cell permeability of this molecule. In the experiment, **13** can diffuse freely into the cells, which is very helpful for the drug-forming properties of the molecule. The two molecules have performed well on the affinity of the PIF-pocket. However, after obtaining the co-crystal structures of these two molecules with PDK1, it is found that the two molecules still have a lot of space for improvement (Figure 12(A) and Figure 12(B)). The sulphur atom on the thiophene at the right end was originally expected to possess an equivalent property to **12**, which could interact with residue Thr148. Therefore, if the sulphur atom on the five-membered ring can be transferred to the six-membered ring, the affinity may be optimised. In addition, in the modification of **3** to **4**, the chlorine atom at the left end can be attempted to be replaced by the trifluoromethyl group. When comparing the binding modes of **13** and **14**, **14** has an extra hydrogen bonding interaction with the residue Thr128 at the helix αC, which may play a role in the more activation of PDK1 by **14** than **13**. Finally, the conformational changes of the helix B at the allosteric PIF-pocket and the ATP molecule induced by both **13** and **14** (Figure 12(C) and Figure 12(D)) are similar to those by **11** and **12** (Figure 11(C) and Figure 11(D)).

### 4.5. Benzimidazole derivatives

Rettenmaier *et al.* had tried to use a structure-based virtual screening which preformed against both crystal structures and comparative models to identify ligands bound to the PIF-pocket of PDK1^98^. Based on the crystal structure of PDK1-**2** complex, a group of six structural models of the PIF-pocket were created. A chemical library of 6,300 property-matched molecules which are commercially available was generated. These molecules were docked against all six structural models for virtual screening. Then the selected molecules were tested by FP competitive binding assay to identify whether the hits were bound to the PIF-pocket of PDK1. Compound 1 (**15**, Figure 7) with an EC_50_ value of ∼40 μM and compound 3 (**16**, Figure 7) with an EC_50_ value of ∼50 μM were identified, respectively. Furthermore, in order to optimise these two molecules, 518 commercially available analogues were extracted from the ZINC database by means of analogue-by-catalogue searching. Finally, 15 analogues were selected based on the scoring equal to or even better the two parent molecules. Among these molecules, RF4 (**17**, Figure 7) is the most potent compound with an EC_50_ value of ∼2 μM.

**Figure 13.**
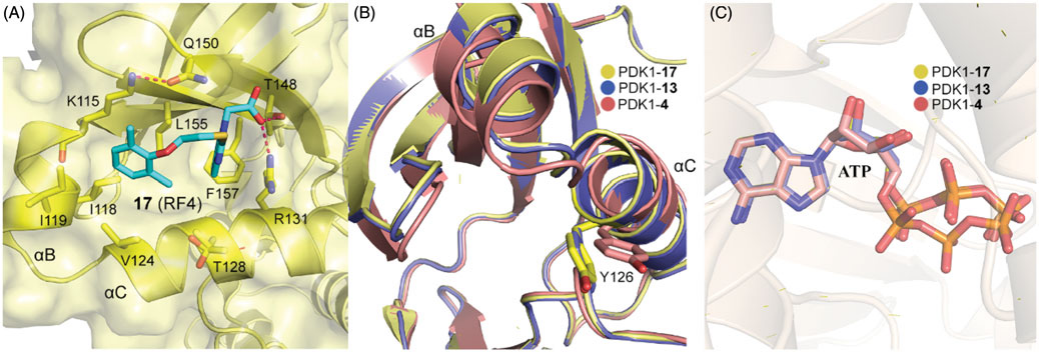
(A) Cartoon/surface representations of the interaction between **17** with the PIF-pocket of PDK1 (pale yellow; PDB ID: 4XX9). (B) Comparison of the helix αB and helix αC of PDK1-**17** complex, PDK1-**13** complex (slate) and PDK1-**4** complex (salmon). (C) Comparison of the ATP of PDK1-**17** complex, PDK1-**13** complex, and PDK1-**4** complex.

The requirements for virtual screening are based on existing models, including the formation of two hydrogen bonds with residues Arg131 and Thr148 and occupying the hydrophobic sub-pockets. It is noteworthy that the 2,6-dimethyl substituted benzene ring of **17** packed tightly into the hydrophobic sub-pocket lined by residues Ile119 and Leu155 of PDK1 (Figure 13(A)), indicating that the 2,4-dimethyl group of **15** was not perfect spatially. This is beneficial for the optimisation of the left benzene ring of **4** or **12**. In addition, **13** and **17** have approximate activation effectiveness, which are quite different from **4**. This phenomenon is consistent with the conformational changes of the helix αB, helix αC and ATP binding site (Figure 13(B) and Figure 13(C)).

### 4.6. Oxypyridine derivatives

**Figure 14.**
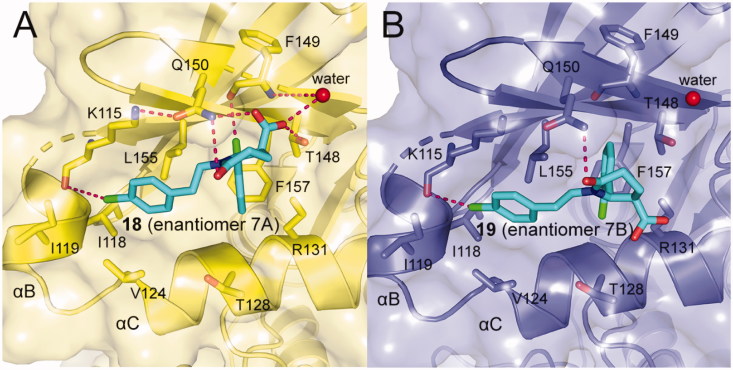
(A) Cartoon/surface representations of the interaction between **18** with the PIF-pocket of PDK1 (pale yellow; PDB ID: 5ACK). (B) Cartoon/surface representations of the interaction between **19** with the PIF-pocket of PDK1 (slate; PDB ID: 5ACK).

**Figure 15.**
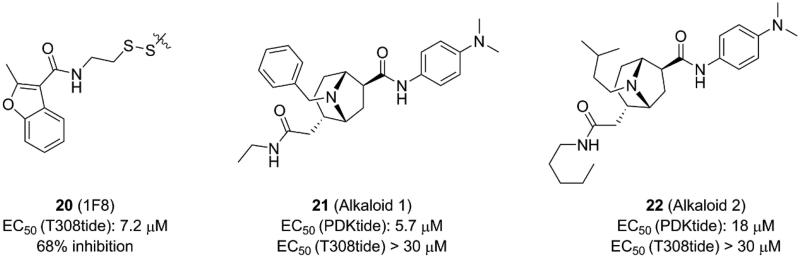
Structure and biochemical characterisation of the allosteric inhibitors directed to the PIF-pocket of PDK1. EC50 (T308tide): compound concentration is required for 50% of maximum effect. EC50 (PDKtide): compound concentration is required to result in 50% displacement of the PDKtide from the PIF-pocket or ATP site in an AlphaScreen interaction-displacement assay.

ANCHOR.QUERY is a method that efficiently allows to obtain active molecules from the known molecule by combination of computation and synthesis^99^. The molecules acquired through this approach can be synthesised in one step by multicomponent reaction (MCR) chemistry. Based on above advantages, Kroon *et al.* applied ANCHOR.QUERY approach for the new scaffolds that bind to the allosteric PIF-pocket of PDK1^100^. The starting point of their investigation was based on the cocrystal structure of PDK1-**2** complex. Through virtual screening and following SAR research, the racemic compound **7** was synthesised. The ability of the racemic to disrupt the PDK1-PIFtide interaction was different, with IC_50_ values of 7.0 μΜ for enantiomer **7 **A (**18**, Figure 8) and 15 μM for enantiomer **7**B (**19**, Figure 8), respectively. In both molecules, the 2-chlorophenyl substituent that acts as an anchor is located in the deep PIF-pocket, but shows a different orientation (Figure 14(A) and Figure 14(B)). In the structure of **18**-bound PDK1, the 2-chlorophenyl substituent forms a short contact with the residue Phe149, while in the structure of **19**-bound PDK1 it turns about 180°, with no contacts with Phe149. In addition, the extra interactions formed by the carboxylic acid group and the amide group of **18** can explain the higher affinity of **18** than **19**.

## 5. Allosteric inhibitors of PDK1

Although past research has focussed on the development of allosteric activators, a few allosteric inhibitors have also been discovered. We try to explain the inhibition mechanism to better understand the activation mechanism.

### 5.1. Disulphide derivatives

**Figure 16.**
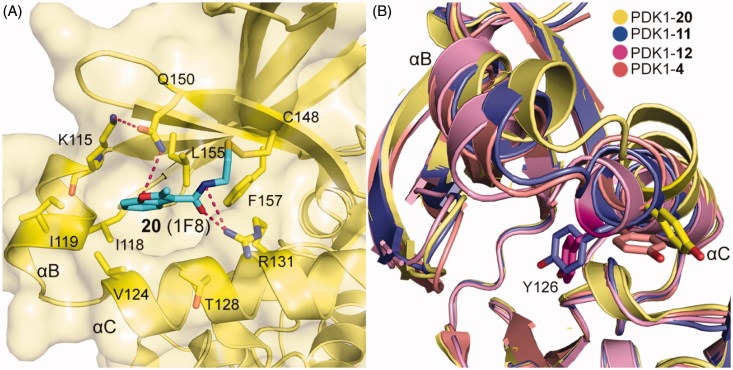
(A) Cartoon/surface representations of the interaction between **20** with the PIF-pocket of PDK1 (pale yellow; PDB ID: 3ORX). (B) Comparison of the helix αB and helix αC of PDK1-**20** complex (pale yellow), PDK1-**11** complex (slate), PDK1-**12** complex (pink) and PDK1-**4** complex (salmon).

By means of the disulphide trapping method, Sadowsky *et al*. also unexpectedly discovered an allosteric inhibitor of PDK1, 1F8 (**20**, Figure 15), which inhibited the activation of PDK1^T148C^ by 68% and with an EC_50_ value of ∼7.2 μM. Like other disulphide derivatives such as **11** and **12**, the affinity of **20** toward the PIF-pocket also primarily relies on the disulphide bond formed with the mutated residue Cys148 (Figure 16(A)). The difference is that the hydrogen bonding interactions of **20** with residues Arg131 and Gln150 are established by the amide group and the oxygen atom on the furan ring, respectively. The allosteric inhibition mechanism of **20** can be inferred by the displacements of helices αB and αC (Figure 16(B)). From **12**, **4**, **11** to **20**, the activity of PDK1 bound to these allosteric small molecules were 630%, 550%, 390%, and 32%. In this process, the position of the helix αB rises in turn. Unfortunately, the obvious rule was not observed between the position of the key helix αC and kinase activity. But the residue Tyr126 on the helix αC gradually moves away from the ATP binding site as the PDK1 activity decreases. It is generally speculated that the allosteric inhibition of PDK1 is caused by the helices αB and αC being away from the active site.

### 5.2. Alkaloid derivatives

Bisubstrate ligand approach, characterised by design of bisubstrate analogues binding to two ligand-binding sites, is promising for developing efficient modulators of protein kinases with multiple binding sites^101^^,^^102^. The three ligand-binding sites possessed by PDK1 were known according to previous studies. Based on the above evidence, Bobkova *et al.* developed a sensitive ultrahigh throughput enzymatic assay to identify modulators of PDK1^103^. They designed a biotinylated fusion peptide that could bind to both ATP binding site and PIF-pocket. This biotinylated fusion peptide was used as the substrate for phosphorylation by PDK1, which allowed them to identify ATP-competitive inhibitors and allosteric modulators of PIF-pocket. After screening over a million of molecules library, the specificity of molecules binding to PIF-pocket was evaluated by thermal shift binding assay and AKT-tide enzymatic assay. Then, alkaloid 1 (**21**, Figure 15) with an EC_50_ value of ∼5.7 μM and alkaloid 2 (**22**, Figure 15) with an EC_50_ value of ∼18 μM were identified, respectively. They exhibited remarkable selectivity against other kinases, but the affinities were very low and showed unmeasurable effect by the AKT-tide enzymatic assay.

To explore the possible interaction of alkaloid derivatives with PDK1, **21** was docked into the PIF-pocket of PDK1. The binding mode indicated that the interactions were dominated by hydrophobic interactions between the **21** and the PIF-pocket of PDK1. The dimethylphenylamine group of **21** occupied a hydrophobic pocket outlined by residues Val127 and Phe157. Additionally, the benzene ring occupied an adjacent hydrophobic site formed by residues Ile118, Ile119, Val124, Val127, and Leu155. However, **21** does not contain a carboxyl moiety, thus it is incapable to form the ionic interaction with residue Arg131 from the PIF-pocket and to further induce conformational changes resembled other allosteric activators of PDK1. The different interactions between alkaloid derivatives with hydrophobic groove may be responsible for the inhibition of PDK1 by such small molecules. Obviously, although **21** and **20** are both allosteric inhibitors of PDK1, their inhibitory mechanisms at the PIF-pocket may be different. Further studies of the allosteric inhibition mechanism of PDK1 by **21** require co-crystallization of this molecule with PDK1.

## 6. Conclusions

The PIF-pocket was initially found to recruit and phosphorylate downstream kinases, and further studies showed that the HM peptides of downstream kinases bound to the PIF-pocket allosterically stimulated the intrinsic activity of PDK1. Therefore, allosteric modulators can be designed to mimic the HM peptide of downstream kinases by attached to the PIF-pocket. To date, seven classes of PDK1 allosteric modulators have been reported based on the similarity of chemical structure, including diary carboxylic acid derivatives, benzoazepin-2-one derivatives, disulphide derivatives, diaryl sulphonamide derivatives, benzimidazole derivatives, oxypyridine derivatives, and alkaloid derivatives. The vast majority of modulators are allosteric activators that activate PDK1 catalytic activity when they are bound to the PIF-pocket, whereas the allosteric inhibitors can also be observed such as **20** that inhibited PDK1 activity. The binding of allosteric activators to the PIF-pocket can stabilise the closure between the PIF-pocket and the leaf, which further stabilised the DFG motif, the activation loop, and the ATP binding site, leading to the stabilisation of catalytic PDK1 conformation. However, allosteric inhibitors binding to the PIF-pocket caused the realignment of helices αB and αC in the inactive conformation, leading to the inhibition of PDK1 activity. Among the reported PDK1 allosteric modulators, **4**, **13**, and **17** showed good binding affinities to the PDK1. Comparison of the three compounds reveals that their interactions with the PIF-pocket are similar. They have two aromatic rings at a distance from each other that occupy the hydrophobic part of the PIF-pocket and are engaged in hydrophobic interactions. There is a fatty chain linking the two aromatic rings that provides a flexible effect. The carbon on the fatty chain can be substituted with an acyl group that can form hydrogen bonds with the residues from the PIF-pocket. Therefore, it is feasible that the future design of PDK1 allosteric modulators may retain the carboxylic acid moiety, the two aromatic rings, and the fatty chain.
